# Only zinc (Zn) among micronutrients is an important predictor of grassland production

**DOI:** 10.3389/fpls.2022.962513

**Published:** 2022-07-22

**Authors:** Congcong Liu, Meng Xu, Ying Li, Nianpeng He

**Affiliations:** ^1^Key Laboratory of Ecology and Environment in Minority Areas (Minzu University of China), National Ethnic Affairs Commission, Beijing, China; ^2^College of Life and Environmental Sciences, Minzu University of China, Beijing, China; ^3^Key Laboratory of Ecosystem Network Observation and Modeling, Institute of Geographic Sciences and Natural Resources Research, Chinese Academy of Sciences, Beijing, China; ^4^Key Laboratory of Prevention and Control for Aquatic Invasive Alien Species, Pearl River Fisheries Research Institute, Chinese Academy of Fishery Sciences, Ministry of Agriculture and Rural Affairs, Guangzhou, China; ^5^College of Grassland Science, Beijing Forestry University, Beijing, China

**Keywords:** biomass production, micronutrients, zinc, iron, model selection

## Introduction

The effects of climatic factors and soil nutrients in the determination of plant biomass production have long been of central interest to ecologists (Huxman et al., 2004; Fay et al., 2015). Recently, Radujković et al. (2021) found that soil micronutrients (particularly Zn and Fe) were important predictors of aboveground biomass production (*Biomass*). In their analyses, the combination of atmospheric factors that best explained the variation in *Biomass* was selected first; then, soil physicochemical properties, C-N-P nutrients, and other nutrients (K, Ca, Mg, Na, S, Zn, Fe, B, Cu, and Mn) were sequentially added to construct structural equation models (SEMs). However, the sequence of nutrient variable introduced to the model matters, and consequently influence the construction of the final best model. More importantly, although separately adding Zn and Fe to the SEM explained additional variations in *Biomass*, they grouped Zn and Fe into one composite variable and argued that both micronutrients were important predictors of *Biomass* given the significant effect of the composite variable. However, they did not examine whether it was appropriate to simultaneously include both Zn and Fe in the model. Here, we re-analyzed the dataset using multiple statistical methods and revealed that the SEM incorporating both micronutrients (including both Zn and Fe) as was done by the original paper was not the best model.

## Data analysis

**Figure 1:**
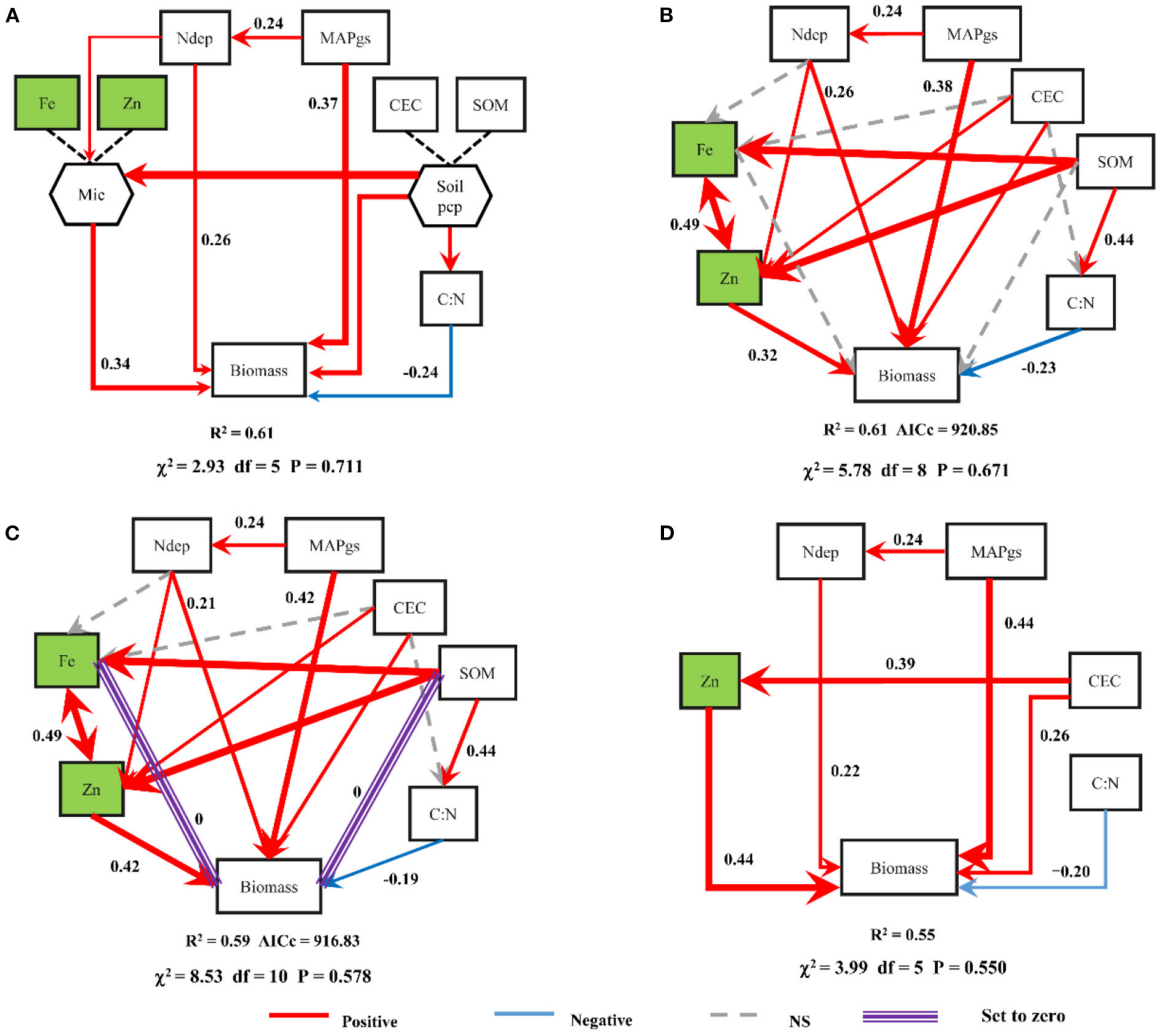
Comparison of structural equation models (SEMs). Panel **(A)**, the SEM of Radujković et al. (2021); Panel **(B)**, Radujković's (2021) SEM without composite variables; Panel **(C)** shows that the direct effects of both Fe and SOM on *Biomass* were set to zero. Panel **(D)**, our best SEM. Numbers along the arrows, and the width of the arrows, indicate standardized path coefficients. MAPgs, mean annual precipitation during the growing season; Ndep, total atmospheric inorganic nitrogen deposition; Fe, extractable iron; Zn, extractable zinc; CEC, cation exchange capacity; SOM, soil organic matter; C:N, carbon-to-nitrogen ratio; Biomass, standing crop (live biomass and recently senescent material) at the peak of the growing season.

In the final model of Radujković et al. (2021) (Figure 1A), seven environmental variables were used to explain the variation in *Biomass*. We first used these seven

variables to fit a multiple linear model: **lm (*Biomass* ~ MAPgs + Ndep + CEC + SOM + C:N + Fe + Zn)**. We found that Fe and SOM had no significant effects on *Biomass*. To further demonstrate this, we used these seven variables to construct an SEM. This SEM was similar to Radujković et al. (2021) final SEM but without composite variables, which showed that Fe and SOM had no significant direct effects on *Biomass* (Figure 1B). After removing these two paths from the model, we found that the SEM was significantly improved, with a lower AICc value (Figure 1C). These results indicate that the final SEM of Radujković et al. (2021) is not the best model to explain the variation in grassland production. Finally, through model comparison and optimization (the R code is provided in the Supplementary Material), we obtained the best SEM to understand the key factors determining grassland production (Figure 1D). Different from their opinion that their results that zinc (Zn) and iron (Fe) were predictors of grassland production, our results indicated that only zinc (Zn) among micronutrients is a significant predictor.

In addition, we used another method to construct a SEM to explain the factors underlying the variation in *Biomass*. Before SEM construction, we performed automated model selection using *glmulti* (Calcagno and De Mazancourt, 2010) based on AICc to determine the best combination of all environmental variables initially used in Radujković et al. (2021). This method allowed us to perform model selection by creating a set of models with all possible combinations of initial variables and sorting them according to the AICc. Five models with ΔAICc <1 were selected (Table 1). In these models, Zn was always retained, and Fe was always excluded. Based on the variables selected by these five linear models, we constructed five SEMs, of which three were consistent with our previous SEM (Figure 1D).

**Table 1:** The best models explained the variation in biomass.

**Model**	**AICc**	*R* ^2^
Biomass ~MAPgs + Ndep + C + N + CN + Na + Zn + SOM	158.4416	0.6139
Biomass ~MAPgs + Ndep + CN + Zn + CEC + pH^ns^	158.5608	0.5834
Biomass ~MAPgs + Ndep + CN + Zn + CEC	159.2411	0.5644
Biomass ~ MAPgs + Ndep + C + N + CN + Na + Zn + SOM+ P^ns^	159.2454	0.6242
Biomass ~MAPgs + Ndep + CN + Zn + CEC + SOM^ns^	159.3996	0.5785

*Automated model selection using glmulti based on AICc, was performed to determine the combination of environmental factors. In multiple linear regression, non-significant environmental factors were labeled with “ns”. Based on these five multiple linear regression models, we constructed five structural equation models (SEMs). In the process of SEM optimization, the final results of three SEMs (corresponding to the bold models) were the SEM shown in Figure 1D. MAPgs: mean annual precipitation during the growing season; Ndep, total atmospheric inorganic nitrogen deposition; Fe, extractable iron; Zn, extractable zinc; Na, extractable sodium; CEC, cation exchange capacity; SOM, soil organic matter; C, total soil carbon; N, total soil nitrogen; CN, carbon-to-nitrogen ratio; P, extractable phosphorus; pH, soil pH; Biomass: standing crop (live biomass and recently senescent material) at the peak of the growing season*.

## Discussion

In summary, we demonstrated that the SEM of Radujković et al. (2021) was not the best model for understanding the predictors of grassland production and that Fe was not a key soil micronutrient for predicting *Biomass* in their dataset. Even though it seems that the difference between Radujković's (2021) results and ours is minor, we argue that it should be highlighted. Our results have at least two important implications. First, the appropriate statistical procedures are the premise of sound scientific finding, and our study provides a paradigm for future SEM construction and optimization. Second, their study may motivate more experimental or observational studies focusing on soil Zn and Fe in the future, but our results indicate that, under the limited experimental resources, only Zn should be a priority for understanding the effects of soil micronutrients on grassland production.

## Author contributions

CL and YL conceived of the comment. CL, MX, and NH performed statistical analyses. CL and YL drafted the manuscript. All authors contributed to the article and approved the submitted version.

## Funding

This work was supported by National Natural Science Foundation of China (31988102, 42141004, and 32171544), the National Science and Technology Basic Resources Survey Program of China (2019FY101300), and the fellowship of China Postdoctoral Science Foundation (2020M680663 and 2021M693147).

## Conflict of interest

The authors declare that the research was conducted in the absence of any commercial or financial relationships that could be construed as a potential conflict of interest.

## Publisher's note

All claims expressed in this article are solely those of the authors and do not necessarily represent those of their affiliated organizations, or those of the publisher, the editors and the reviewers. Any product that may be evaluated in this article, or claim that may be made by its manufacturer, is not guaranteed or endorsed by the publisher.
